# NMDAR1 autoantibodies amplify behavioral phenotypes of genetic white matter inflammation: a mild encephalitis model with neuropsychiatric relevance

**DOI:** 10.1038/s41380-021-01392-8

**Published:** 2021-12-06

**Authors:** Sahab Arinrad, Justus B. H. Wilke, Anna Seelbach, José Doeren, Martin Hindermann, Umer Javed Butt, Agnes A. Steixner-Kumar, Lena Spieth, Anja Ronnenberg, Hong Pan, Stefan A. Berghoff, Michael Hollmann, Fred Lühder, Klaus-Armin Nave, Karl Bechter, Hannelore Ehrenreich

**Affiliations:** 1 Clinical Neuroscience, Max Planck Institute of Experimental Medicine, Göttingen, Germany; 2 Department of Neurogenetics, Max Planck Institute of Experimental Medicine, Göttingen, Germany; 3 Department of Biochemistry I—Receptor Biochemistry, Ruhr University, Bochum, Germany; 4 Institute of Neuroimmunology and Multiple Sclerosis Research, University Medical Center, Göttingen, Germany; 5 Department of Psychiatry and Psychotherapy II, Ulm University, Günzburg, Germany

**Keywords:** Neuroscience, Psychiatric disorders

## Abstract

Encephalitis has an estimated prevalence of ≤0.01%. Even with extensive diagnostic work-up, an infectious etiology is identified or suspected in <50% of cases, suggesting a role for etiologically unclear, noninfectious processes. Mild encephalitis runs frequently unnoticed, despite slight neuroinflammation detectable postmortem in many neuropsychiatric illnesses. A widely unexplored field in humans, though clearly documented in rodents, is genetic brain inflammation, particularly that associated with myelin abnormalities, inducing primary white matter encephalitis. We hypothesized that “autoimmune encephalitides” may result from any brain inflammation concurring with the presence of brain antigen-directed autoantibodies, e.g., against N-methyl-D-aspartate-receptor NR1 (NMDAR1-AB), which are not causal of, but may considerably shape the encephalitis phenotype. We therefore immunized young female *Cnp*^−/−^ mice lacking the structural myelin protein 2′-3′-cyclic nucleotide 3′-phosphodiesterase (Cnp) with a “cocktail” of NMDAR1 peptides. *Cnp*^−/−^ mice exhibit early low-grade inflammation of white matter tracts and blood–brain barrier disruption. Our novel mental-time-travel test disclosed that *Cnp*^−/−^ mice are compromised in what–where–when orientation, but this episodic memory readout was not further deteriorated by NMDAR1-AB. In contrast, comparing wild-type and *Cnp*^−/−^ mice without/with NMDAR1-AB regarding hippocampal learning/memory and motor balance/coordination revealed distinct stair patterns of behavioral pathology. To elucidate a potential contribution of oligodendroglial NMDAR downregulation to NMDAR1-AB effects, we generated conditional NR1 knockout mice. These mice displayed normal Morris water maze and mental-time-travel, but beam balance performance was similar to immunized *Cnp*^−/−^. Immunohistochemistry confirmed neuroinflammation/neurodegeneration in *Cnp*^−/−^ mice, yet without add-on effect of NMDAR1-AB. To conclude, genetic brain inflammation may explain an encephalitic component underlying autoimmune conditions.

## Introduction

The discovery in 2007/2008 of autoantibodies directed against the NMDA receptor subunit NR1 (NMDAR1-AB = GluN1-AB) in the context of a paraneoplastic autoimmune disease, has stimulated extensive interest of clinicians and clinical researchers worldwide [1, 2]. As a consequence, diagnoses of autoimmune conditions underlying neuropsychiatric disorders of different severity and highly variable presentation began to boom [3–5]. Etiology and pathogenesis of “NMDAR encephalitis,” however, are still as ambiguous as the role of NMDAR1-AB in this condition. In fact, NMDAR1-AB, reaching the brain from the circulation via a compromised blood–brain barrier (BBB), or being produced intrathecally, act as NMDAR antagonists, i.e., they produce ketamine-like phenotypical effects [6, 7]. This is mechanistically accomplished by internalization of the receptor, resulting (sub)acutely in lower surface expression of neuronal NMDAR upon NMDAR1-AB binding [8–12]. Together with probably diverse local access to the brain [13], it matches well the broad spectrum and inconstant intensity of psychopathological and neurological symptoms, ranging from psychosis, cognitive decline and extrapyramidal signs to motor dysfunction, autonomic dysregulation and epileptic seizures [1, 2]. At certainly lower amounts and dependent on the condition, NMDAR1-AB may also act as antidepressants [14] or in certain situations even behave like a double-edged sword, e.g., in ischemic stroke, where they can protect acutely from evolution of lesion size [15], but upon long-term exposure seem to contribute to poststroke dementia [16].

Notably, numerous brain-directed AB—apart from NMDAR1-AB—have been reported in serum of healthy humans and of various other mammalian species, likely constituting the preexisting physiological “autoimmune repertoire.” These AB do have potential functionality and pathogenicity [3, 13, 17–19]. Regarding NMDAR1-AB, all immunoglobulin (Ig) classes are highly seroprevalent across mammals, with multiple possible inducers or boosters identified [2, 11, 14, 20–22]. But neither the “rules” of their induction nor the potentially different (patho)physiological significance of the various Ig classes has been elucidated yet, even though the class distribution of NMDAR1-AB is highly significantly predicted by the extracellular position of the antigen [23].

Importantly, a causal input of NMDAR1-AB themselves concerning encephalitis induction has not been convincingly demonstrated yet [24]. At least mild neuroinflammation [25, 26], if not obvious viral encephalitis caused by infections like herpes or influenza [11, 20–22], most likely prevail already during early stages of brain autoimmune disease. This might ultimately explain the encephalitic component of these conditions, and seamlessly supports Karl Bechter’s mild encephalitis hypothesis of mental illness [25, 26]. Also the white matter damage described in “NMDAR encephalitis” patients with incomplete recovery blends into this view, highlighting the heterogeneity of underlying causes [27]. Overall, origins of encephalitides amount to 20–50% documented viral infections [28–30]. The remaining causes are to a large degree unknown, including the roots of mild encephalitis, running mostly undiagnosed. Of particular interest in this regard are genetic reasons of neuroinflammation, mainly those associated with myelin abnormalities leading to mild primary white matter encephalitis [31–33].

We note that already the normal aging process is associated with slightly increased brain inflammation, characterized by, e.g., enhanced levels of proinflammatory cytokines, higher microglial numbers and heightened reactivity [31, 34–36]. In major psychiatric disorders like schizophrenia and depression, low-grade inflammation constitutes a crucial mechanism in the final common disease pathway (reviewed in, e.g., [37, 38]), that has been linked to white matter abnormalities, as documented by postmortem studies. *CNP* (2′,3′-cyclic nucleotide 3′-phosphodiesterase) is among the oligodendrocyte/myelin-associated genes most robustly reduced on mRNA and protein level in these brains [39–41]. In fact, Cnp/CNP is a structural protein, present in noncompacted myelin and accounting for about 4% of total central nervous system myelin proteins [42]. Null mutant (*Cnp*^−/−^) mice constitute a translationally interesting model of genetically induced, progressive brain inflammation [43]. Starting already at a few weeks of age, these animals develop behavioral abnormalities, low-grade white matter inflammation and progressive neurodegeneration [32, 44]. Analogously, in humans with a *CNP* loss-of-function allele (*CNP* single-nucleotide polymorphism, SNP: rs2070106-AA), diffusion tensor imaging points to axonal loss in the frontal corpus callosum [31], and white matter hyperintensities in rs2070106-AA carriers indicate mild signs of neuroinflammation and demyelination [32]. These findings suggest that *CNP* reduction (and the herewith associated inflammation) might be critical in a more general disease process and that the potential role of this protein is not restricted to a single neuropsychiatric diagnostic category but of global relevance for severe mental disorders.

Here, we immunized young female *Cnp*^−/−^ mice with a cocktail of 4 NMDAR peptides [9, 24] to generate a novel autoimmune model based on preexisting white matter encephalitis. This cocktail includes a peptide covering the NMDAR1-N368/G369 region, claimed to be pathognomonic for NMDAR1-AB encephalitis [45], and most importantly, it induces functionally highly active NMDAR1-AB, leading to psychosis-like symptoms in mice with compromised BBB [9]. We report that NMDAR1-AB can substantially contribute to the behavioral abnormalities of an underlying white matter encephalitis, reflected by striking stair patterns of pathology. As indicated by additional behavioral phenotyping of conditional NR1 mutant mice, part of these behavioral NMDAR1-AB effects might be due to downregulation not only of neuronal, but also of oligodendroglial NMDAR.

## Materials and methods

### Mice

All animal experiments were conducted in accordance with the local authorities, i.e., the Animal Care and Use Committee (Niedersächsisches Landesamt für Verbraucherschutz und Lebensmittelsicherheit, LAVES). Sample sizes were based on previous experience under consideration of the RRR principle and adjusted to technical limitations (e.g., maximum of 16 mice per IntelliCage). All experiments were performed by investigators unaware of group assignment (“fully blinded”). Animals were kept under standard laboratory conditions (20–22 °C, 12 h light/dark cycle, lights off at 6 p.m.) in conventional type IV (*n* = 13–16 per cage) or type II cages (*n* = 3–5 per cage) (Tecniplast, Hohenpeißenberg, Germany) and maintained within ventilated cabinets (Scantainers, Scanbur Karlsunde, Denmark), separated by gender and with access to food (Sniff Spezialdiäten, Bad Sodenberg, Germany) and water ad libitum. Genotyping was carried out as previously described for *Cnp* [31, 43] and *NR1*^*flox/flox*^ [46, 47].

### Experimental cohorts

1. Immunization cohort: Female *Cnp*^−/−^ (KO, *n* = 26) mice on a C57Bl/6 background along with their wild-type (WT, *n* = 14) littermates were immunized at age 8 weeks with either a cocktail of four GluN1 extracellular peptides (GluN1_35-53_, GluN1_361-376_, GluN1_385-399_, and GluN1_660-811_, coupled to keyhole limpet hemocyanin; Synaptic Systems, Göttingen, Germany) plus chicken ovalbumin (WT, *n* = 14, KO, *n* = 13) or ovalbumin only (KO, *n* = 13) (“OVA,” A5503, Sigma-Aldrich, Taufkirchen, Germany) as previously described [9, 24].
2. Oligodendroglial NR1 cKO cohorts: Female *CnpCre*^*+/*−^*:NR1*^*flox/flox*^ (cKO, *n* = 16) mice and their *NR1*^*flox/flox*^ (*n* = 16) littermate controls were employed to elucidate the effect of NR1 deficiency on behavior between 3 and 11 months of age. A separate cohort of aged (12–14 months) female *CnpCre*^+/−^*:NR1*^*flox/flox*^ (*n* = 10) and *NR1*^*flox/flox*^ controls (*n* = 12) was used to confirm findings.
3. *Cnp* deficiency control cohorts: *Cnp*^+/−^ (*n* = 7) and WT (*n* = 9) littermates were used at age 12 months as controls to separate *Cnp* heterozygosity effects from oligodendroglial NR1 cKO. For BBB integrity analysis, male WT (*n* = 4) and *Cnp*^−/−^ (*n* = 5) mice, aged 18 weeks, were used.

### Transponder placement

Mice were anesthetized by intraperitoneal injections of 24 µL 2,2,2,-tribromoethanol (1.36%; T48402, Sigma) in ddH_2_O/g body weight (Avertin). ISO standard transponders (8.5 mm length, 1.2 mm diameter, PM162-8) were implanted below the skin of the neck to enable experimenter-independent phenotyping of mice in IntelliCages^®^ (TSE Systems, Bad Homburg, Germany). Mice were introduced to the IntelliCages on the following day.

### Behavioral characterization

For detailed information on order of testing in all cohorts, we refer to the respective figures.

IntelliCage-based behavioral phenotyping and mental-time-travel (MTT): The IntelliCage battery was carried out as detailed before [48], covering cognitive functions like place learning, reversal learning, and sucrose preference for assessment of potential anhedonia. Subsequently, higher-order cognition was tested over 9 days using our novel IntelliCage-based MTT paradigm. For this, mice receive tap water via a nose poke at first from all four corners. Starting with the second experimental day, access to drinking water is limited to a brief time window only during the active phase of the mice (6–8 p.m.). In one of the corners, mice receive a 1.5 bar air puff as negative reinforcement (day I). The individually assigned “punished” corner changes for each mouse on a daily basis following a distinct pattern, namely diametrically opposed to the first punished corner (day II), horizontally opposed (day III) and again diametrically opposed (day IV) (training cycle). This pattern is then repeated for a second round (days V–VIII) and the preference (% visits) to each corner on each day of the second round is used to assess MTT abilities (assessment cycle). Each corner on each day is considered either currently (=0 days after punishment), recently (=1 day after punishment), intermediately (=2 days after punishment) or longer ago punished (=3 days after punishment). The average number of visits to each of these corners throughout the assessment cycle is calculated and used for statistical analyses. The steepness of the curve, best expressed as trend line, reflects the quality of MTT performance.

### Spatial memory and reversal learning

To assess hippocampus-dependent cognitive functions such as spatial learning and memory, we conducted the classical Morris water maze (MWM) as previously described [49–51]. Immunized mice were tested at 50–64 days post-immunization (DPI); cKO mice at the age of 3 months, including a subsequent reversal learning period followed by a second probe trial.

Motor performance, motor learning and coordination: Motor coordination was analyzed by beam balance as previously described [31, 50]. Motor performance and learning was tested using rotarod as previously described [31, 49]. Female cKO mice, having revealed a pathological rotarod phenotype, as well as *Cnp*^+/−^ mice as controls were exposed to 4 h of voluntary running on complex running wheels (CRW) as previously described [52, 53] to further assess motor-cognitive performance.

### Blood sampling and NMDAR1-AB determination

Blood samples were collected from the retro-orbital sinus prior to immunization, before transponder placement (29 DPI) and before perfusion (~3 months post-immunization). EDTA plasma aliquots were stored at −80 °C. Specific antigen ELISA was performed as previously described [9, 24] using plasma diluted 1:1000 in phosphate buffered saline (PBS), to confirm successful immunization and persistence of NMDAR1-AB. To determine NMDAR1-AB IgG formation against full-length GluN1, a commercially available cell-based assay comprising *Grin1*-transfected and control-transfected HEK293 cells (FB 112d-1010-51, EUROIMMUN, Lübeck, Germany) was used as previously described [24]. Terminal plasma samples were diluted 1:100 in 0.2% Tween20/PBS and spiked with a commercial rabbit anti-GluN1 antibody directed against the intracellular C-terminal domain (1:1000, G8913, Sigma). Bound mouse and rabbit IgG were detected with Alexa Fluor 488-labeled anti-mouse-IgG (1:1000, A21202, Thermo Fisher Scientific, Darmstadt, Germany) and Alexa Fluor 647 labeled anti-rabbit IgG antibody (1:1000, A31573, Thermo). Nuclei were stained 10 min at RT with 1 µg/mL 4′,6-diamidino-2-phenylindole in PBS (DAPI, D9542, Sigma). Representative images were acquired on a confocal microscope (LSM 880, Zeiss, Oberkochen, Germany).

### Measurements assessing BBB integrity

BBB integrity was evaluated by quantifying extravasation of two distinct fluorescent tracers (Evans blue and fluorescein) as previously described [9, 54]. In addition, brain water content was determined by comparing wet and dry brain mass.

### Histology

Mice were anesthetized with Avertin and transcardially perfused with Ringer (B. Braun, Melsungen, Germany). Brains were collected and hemispheres separated. One hemisphere was snap-frozen and the other postfixed in 4% formaldehyde/PBS for 12 h, dehydrated in 30% sucrose/PBS for 48 h, embedded in optimal cutting medium (Tissue-Tek, #4583, Sakura, Umkirch, Germany) and frozen on dry ice. Fixed hemispheres were cut into 30 µm coronal sections on a cryostat (CM1950, Leica, Wetzlar, Germany) and stored at −20 °C in cryomedium (25% ethylene glycol/25% glycerol/PBS). Quantifications were performed as described previously [24, 51], using 3–5 regularly spaced sections per mouse (every 150 µm) between Bregma coordinates −1.34 and −2.24 mm. Free-floating frozen sections were blocked and permeabilized for 1 h at RT with 5% normal horse serum (NHS, 26050-088, Thermo) in 0.5% Triton X-100/PBS and incubated overnight at 4 °C with primary antibodies. Following primary antibodies were used: Mouse anti-GFAP (1:500, NCL-GFAP-GA5, Novocastra-Leica, Newcastle upon Tyne, UK), rabbit anti-Iba1 (1:1000, #019-19741, Wako, Neuss, Germany), guinea pig anti-parvalbumin (1:1000, #195004, Synaptic Systems), rat anti-CD3 (1:100, clone 17A2, BioLegend, Koblenz, Germany), rabbit anti-CD19 (1:500, clone D4V4B, Cell Signaling Technologies, Frankfurt am Main, Germany). Subsequently, sections were stained with corresponding fluorescently-labeled secondary antibodies for 2 h at RT. Secondary antibodies included: Alexa Fluor 555 anti-rabbit (1:1000, A21428, Thermo), Alexa Fluor 633 anti-guinea pig (1:1000, A21105, Thermo), Alexa Fluor 647 anti-mouse (1:1000, A31571, Thermo), and Alexa Fluor 647 anti-rat (1:1000, A21247, Thermo). Nuclei were stained for 10 min at RT with 0.2 µg/mL 4′,6-diamidino-2-phenylindole in PBS (DAPI, D9542, Sigma). Sections were mounted on SuperFrost^®^-Plus slides (J1800AMNZ, Thermo) with Aqua-Poly/Mount (#18606, Polysciences, Warrington, PA, USA) and imaged on a confocal laser scanning microscope (LSM 880, Zeiss). For quantification of Iba1 and GFAP, 1 µm thick optical sections of hippocampi and corpora callosa were acquired as tile scans using a 40× oil objective (40×/1.4 NA Plan-APOCHROMAT, #420762-9900, Zeiss). Parvalbumin, CD3 and CD19 stainings were imaged using a 20× air objective (20×/0.8 Plan-APOCHROMAT, #420640-9903, Zeiss) with an optical thickness (Z-resolution) of 2 µm for parvalbumin and 5 µm for CD3 and CD19 stainings. Image acquisition parameters were kept constant within experiments. Quantifications and image processing were performed with FIJI-ImageJ software (Schindelin). Iba1+ cells (mostly microglia), parvalbumin+ cells (inhibitory neurons), CD3+ cells (T cells), and CD19+ cells (B cells) were manually counted. GFAP+ area was quantified densitometrically upon uniform thresholding. Cell counts and GFAP+ area were normalized to quantified areas. Data from 3–5 hippocampi/mouse was averaged. Fluorojade C staining of dying neurons was performed as previously described [51]. Neuronal death was qualitatively evaluated in hippocampus and corpus callosum by a blinded investigator using two sections per mouse. Representative images were acquired as tile scans on a confocal laser scanning microscope (LSM 880, Zeiss). DAB-based APP staining and quantification of APP+ axonal swellings was carried out in corpus callosum and hippocampus (three sections per mouse) as described previously [32], using a monoclonal anti-APP antibody (1:850, MAB348, Merck KGaA, Darmstadt, Germany) in 3% NHS/0.5% Triton X-100 in PBS for 48 h at 4 °C. Biotinylated horse anti-mouse secondary antibody (1:200, Vector Laboratories, Burlingame, CA, USA) in 3% NHS/0.5% Triton X-100 in PBS followed by Vectastain Elite ABC Kit (Vector Laboratories) were applied according to manufacturer’s instructions. Mayer’s hemalum (Merck KGaA, Darmstadt, Germany) served as counterstain of nuclei. Quantifications were conducted with Stereoinvestigator 6.55 software (MicroBrightfield Inc.), using a light microscope (Olympus BX-50, Olympus, Hamburg, Germany), attached to a computer-driven motorized stage and a microfire video camera. Representative images were obtained on a light microscope (Zeiss Imager Z1) with a 40×/NA 1.30 oil objective lens.

### Blood flow cytometry

Per mouse, 50 µL EDTA blood was diluted in 50 µL PBS and overlaid on 100 µL lymphocyte separation medium (1077, PromoCell). After centrifugation, cells were isolated from the interphase, washed and stained for 15 min at 4 °C with the following antibodies: PECy5 anti-CD4 (1:1000, clone H129.19, BioLegend), PECy7 anti-CD8 (1:500, clone 53-6.7, BioLegend), BV510 anti-B220 (1:333, clone RA3-6B2, BioLegend), PerCpCy5.5 anti-CD11b (1:1000, clone M1/70, BioLegend), PE anti-Gr1 (1:1000, clone RB6-8C5, BioLegend), and FITC anti-F4/80 (1:1000, BM8, BioLegend). After staining, cells were washed, suspended in 250 µL PBS containing 2% bovine serum albumin (#8076.3, Roth), and filtered through 40 µm cell strainers. Samples were measured on a FACSAria Sorp (BD). Total cell numbers were determined using forward and side scatter. Frequency of helper T cells (CD4+, CD8−), cytotoxic T cells (CD8+, CD4−), B cells (B220+), macrophages (CD11b+, F4/80+) and neutrophils/monocytes (CD11b+, Gr1+) were determined as percentage of total lymphocytes.

### Statistical analysis

Statistical analyses were performed using Prism software (GraphPad Software, version 9) or R 4.0.5. Data normality was assessed using the Shapiro–Wilk test with an alpha error of 0.05. Dependent on data distribution, two-tailed unpaired Welch’s corrected *t*-test or Mann–Whitney *U* tests were used to compare groups of 2. Similarly, groups of three were compared by Welch’s ANOVA or Kruskal–Wallis test. Linear trends were tested via Jonckheere–Terpstra trend tests. Repeated measure data was analyzed using mixed-model ANOVA. Linear regression analyses of MTT datasets were performed in Prism 9 using least squares regression, a straight line model, and extra sum of squares *F* test to compare slopes. Results are presented as mean ± SEM; *p* values < 0.05 were considered statistically significant.

## Results and discussion

### Immunization of *Cnp*^−/−^ mice, a genetic model of mild primary white matter encephalitis, against NMDAR1

**Fig. 1:**
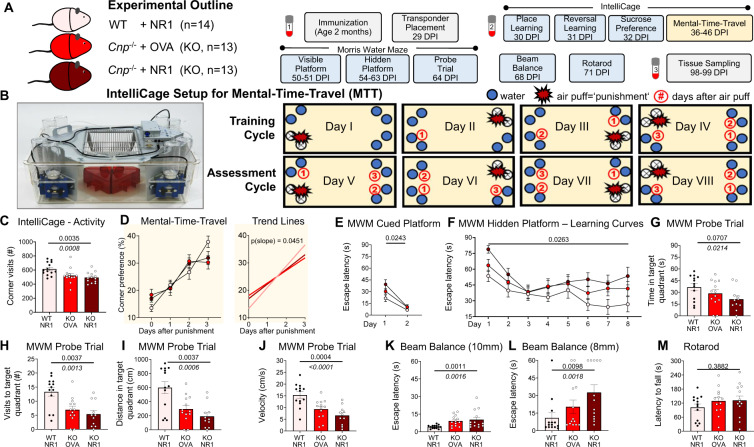
Amplification of pathological behavior in white matter inflammation by high levels of circulating NMDAR1-AB (=GluN1-AB), giving rise to remarkable behavioral stair patterns. **A** Experimental outline of the immunization study using female C57Bl/6 WT (*n* = 14) and *Cnp*^−/−^ (KO, *n* = 2 × 13) mice. Numbered vials (1–3; compare Fig. 2) represent time points of blood sampling. DPI, days post-immunization; NR1 cocktail of four GluN1 extracellular peptides (GluN1_35-53_, GluN1_361-376_, GluN1_385-399_, and GluN1_660-811_), OVA, ovalbumin. **B** Schematic depiction of the novel IntelliCage-based experimental paradigm of mental-time-travel (MTT): Illustrating photograph of the IntelliCage kindly provided by TSE Systems (Berlin, Germany); top row (days 1–4) represents the training cycle, bottom row (days 5–8) the assessment cycle, used for statistical analysis; blue circles denote water bottles (two per corner) placed in the corners of the cage; red star-shaped symbol indicates the currently punished (1.5 bar air puff) corner of the cage on the respective experimental day; red encircled numbers refer to the conditioned corners following the order of experienced punishment over test days. **C** Overall activity during IntelliCage-based testing battery assessed by sum of total visits to all corners (#) in 72 h, including place learning, reversal learning, sucrose preference. **D** MTT of WT versus KO expressed as preference to corners (visits in %) that were either currently (0 days after punishment), recently (1 day), intermediately (2 days), or longer ago (3 days) punished. Both KO groups (*n* = 13 each), irrespective of immunization, exhibit reduced MTT abilities compared to WT (*n* = 14), as demonstrated by the trend lines (right), calculated by linear regression (GraphPad Prism 9 Software, San Diego, CA, USA), delineating the different slopes. All slopes were nonzero (*p* < 0.001), showing successful execution and MTT learning. (**E**–**J**) Impaired spatial memory in the Morris water maze (MWM) test shown during cued (**E**) and hidden (**F**) platform training as well as during the probe trial (**G**–**J**). Immunized KO mice exhibit the poorest performance during the probe trial regarding time spent in (**G**), number of visits to (**H**) and distance swum in the target quadrant (**I**), as well as on average velocity (**J**). A total of 13 immunized WT and ovalbumin-treated KO mice were compared to 11 immunized KO mice. (**K**–**M**) Beam balance (motor coordination) testing reveals again stair patterns of performance on 10 mm (**K**) and 8 mm (**L**) diameter beams, while motor performance on the rotarod (**M**) appears unaffected; results of group effects of repeated measure mixed-models ANOVA, Welch’s ANOVA, or Kruskal–Wallis test on top, of Jonckheere–Terpstra trend test in italics underneath; mean ± SEM presented.

Young female *Cnp*^−/−^ mice lacking the structural myelin protein 2′-3′-cyclic nucleotide 3′-phosphodiesterase (Cnp), which is causal of progressive, genetically induced white matter inflammation [43], were immunized at the age of 8 weeks with a cocktail of four GluN1 extracellular peptides (GluN1_35-53_, GluN1_361-376_, GluN1_385-399_, and GluN1_660-811_) [9, 24], including a peptide in the N-terminal domain containing the G7 epitope (N368/G369) [45], versus ovalbumin as control immunization. We used females to account for the 4:1 female/male ratio in human “NMDAR encephalitis” [55]. WT mice receiving the identical cocktail of four GluN1 extracellular peptides served as controls (all same C57BL/6 background, age and gender as *Cnp*^−/−^ mice). Transponders were implanted 4 weeks later and mice went through a series of tests as detailed in Fig. 1A.

### Modeling autoimmune encephalitis: NMDAR1-AB aggravate behavioral consequences of white matter inflammation

Mouse behavior testing was started in an observer-independent setting, namely our previously designed, extensive cognitive, emotional and social phenotyping, using IntelliCages (Fig. 1B; [48]). This setting did not reveal notable differences between groups (Table 1), except for a reduction in the total number of corner visits (assessed over the first three IntelliCage paradigms), resulting in a stair pattern from WT to *Cnp*^−/−^ without and with NMDAR1-AB (Fig. 1C). This paucity of differences in the IntelliCage setup is most likely explained by a relatively low sensitivity or ceiling effects of this paradigm, requiring prominent brain damage to show changes [24]. However, in an attempt to provide more challenging tasks for testing of higher brain functions, including executive performance, we established a novel test, described here for the first time, which measures episodic-like memory in the IntelliCage setup (Fig. 1B). Mice have to show their ability of what–where–when discrimination [56–58]. Indeed, *Cnp*^−/−^ mice with their progressing white matter inflammation reveal compromised MTT capacity. In this task, the presence of NMDAR1-AB had no add-on effect. These results are clearly visualized by the trend lines showing the steepest curve (best MTT performance) in WT controls (Fig. 1D).

**Table 1:** IntelliCage-based cognitive and emotional testing.

(A) Mice immunized with GluN1 extracellular peptides (NR1) or ovalbumin (OVA)								
	Group 1		Group 2		Group 3		Comparison	
	WT + NR1		*Cnp*^−/−^ + OVA		*Cnp*^−/−^ + NR1		Across groups	
	Mean ± SEM	*n*	Mean ± SEM	*n*	Mean ± SEM	*n*	Test	*p* value
IntelliCage-based behavioral test battery								
Place learning − place errors [% of corner visits]	67.89 ± 1.6	14	70.98 ± 1.1	13	71.49 ± 1.1	13	Welch’s ANOVA	0.207
Reversal learning − place errors [% of corner visits]	66.25 ± 1.7	14	70.01 ± 1.7	13	71.18 ± 1.1	13	Kruskal–Wallis	0.087
Sucrose preference − delta [sucrose − water]	15.68 ± 3.5	14	15.38 ± 2.8	13	14.45 ± 2.7	13	Kruskal–Wallis	0.673

(B) Mice with oligodendrocyte-specific deletion of NR1						
	Group 1		Group 2		Comparison	
	*NR1*^*flox/flox*^		*CnpCre*^+/−^*NR1^*flox/flox*^		Group 1 versus Group 2	
	Mean ± SEM	*n*	Mean ± SEM	*n*	Test	*p* value
IntelliCage-based behavioral test battery						
Place learning − place errors [% of corner visits]	68.37 ± 1.2	15	69.70 ± 1.3	14	*t*	0.463
Reversal learning − place errors [% of corner visits]	62.21 ± 1.7	15	68.94 ± 1.7	14	*t*	0.011
Sucrose preference − delta [sucrose − water]	15.03 ± 4.4	15	10.29 ± 2.7	14	*t*	0.373

Similar to the corner visits in the IntelliCage (Fig. 1C), nearly all subsequently conducted conventional tests, including MWM readouts of hippocampal learning and memory, presented with line and bar graphs (Fig. 1 E–J), as well as beam balance performance (Fig. 1K, L) revealed highly significant stair patterns. Just the final test, rotarod, did not show differences between groups, pointing to a widely normal motor performance. In conclusion, these data indicate that in many behavioral paradigms, the presence of NMDAR1-AB aggravated the measured pathology, consistent with a significant autoimmune shaping of the encephalitis phenotype. These findings in white matter inflammation are in interesting contrast to previously modeled gray matter inflammation—where only marginal shaping of the phenotype by NMDAR1-AB was observed [24]. We note, however, that in the gray matter model, pyramidal neurons were induced to express diphtheria toxin, leading to a substantially reduced excitatory neuron number. Therefore, NMDAR were already highly diminished and their downregulation by NMDAR1-AB did not further exacerbate the resulting pathological picture [24].

### Comparable formation and persistence of circulating NMDAR1-AB in all immunized mice

**Fig. 2:**
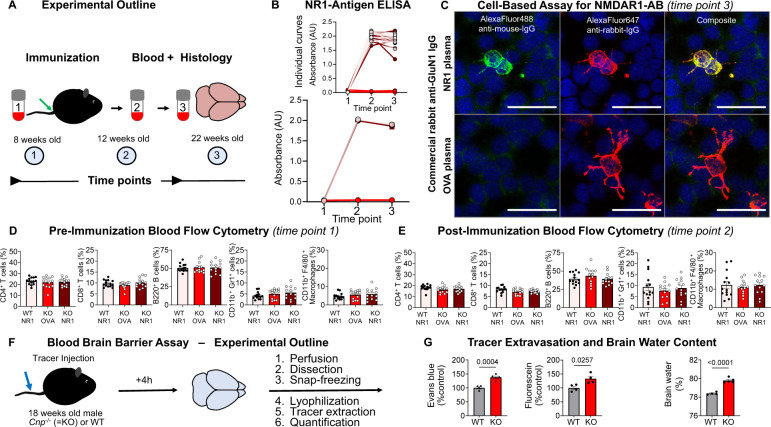
Validation of immunization, analysis of main peripheral immune cell distribution, and assessment of BBB functionality in *Cnp*^−/−^ (KO) and WT mice. **A** Experimental outline depicting time points of blood sampling, immunization, and tissue processing. **B** NR1-antigen ELISA, showing substantial NMDAR1-AB formation at the start of behavioral testing and NMDAR1-AB persistence throughout the experimental period (~3 months); data of 13–14 mice/group; mean ± SEM. **C** Immunocytochemical co-localization of NR1-immunized mouse plasma (1:100, green) with a commercial rabbit GluN1-AB (red) in a cell-based (HEK293T) clinical standard assay for NMDAR1-AB (Euroimmun); plasma (1:100) of OVA-immunized mice did not show specific staining for NMDAR1; GluN1/NR1, glutamate ionotropic receptor NMDA type subunit 1; OVA, ovalbumin. (**D**, **E**) Flow cytometric analysis of peripheral blood immune cells before (**D**) and 1 month after immunization (**E**). Note the physiological peripheral immune cell subsets in all groups, despite white matter inflammation in *Cnp*^−/−^ mice; data of 13–14 mice/group mean ± SEM. **F** Experimental outline for the assessment of blood–brain barrier (BBB) function in 18-week-old male *Cnp*^−/−^ and WT mice. **G** Extravasation of Evans blue and fluorescein into CNS tissue as well as increased brain water content in *Cnp*^−/−^ mice; data of 4–5 mice/group; two-tailed unpaired Welch’s corrected *t*-test or Mann–Whitney *U* tests; mean ± SEM.

Testing the immunization efficiency of our cocktail of four GluN1 extracellular peptides with a respective NR1-antigen ELISA confirmed the presence of high circulating NMDAR1-AB levels in all immunized mice that persisted up to the end of the experiment at age 22 weeks (Fig. 2A, B). Moreover, a cell-based assay using NR1-transfected cells revealed the presence of specific NMDAR1-AB, additionally validated by double-labeling with a commercial rabbit anti-GluN1 IgG (Fig. 2C).

### Peripheral immune cells in post-immunization flow cytometry remain unremarkable despite BBB disruption in *Cnp*^−/−^ mice

Blood flow cytometry at 12 weeks of age, i.e. 4 weeks after immunization, did not show any abnormalities of *Cnp*^−/−^ mice with or without NMDAR1-AB in number and distribution of circulating immune cells compared to WT or pre-immunization state (Fig. 2D, E). In contrast, determination of BBB integrity [9, 24, 54] showed distinctly enhanced tracer extravasation and brain water content (Fig. 2F, G), consistent with a clear BBB disruption, allowing NMDAR1-AB to readily reach the brain. Similar to the observations in gray matter encephalitis after diphtheria toxin-induction [51], the complete lack of peripheral changes in a situation of substantial white matter inflammation, including BBB breakdown and inflammatory degeneration, is intriguing. At the same time it is alarming for clinicians who are not infrequently confronted with the necessity to diagnose or exclude an encephalitis in vivo, particularly upon suddenly occurring behavioral abnormalities in neuropsychiatric practice [51].

### Histological analysis of basic brain inflammation readouts fails to reveal any add-on effect of NMDAR1-AB

**Fig. 3:**
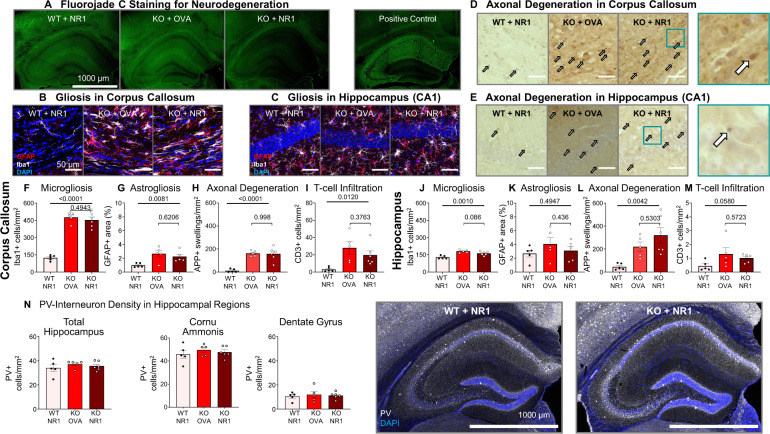
Histological examination of neuroinflammation and neurodegeneration in immunized *Cnp*^−/−^ (KO) and WT mice. **A** Fluorojade C staining as sensitive marker of dying neurons shows absence of neuronal death in all three groups; representative images provided; as positive control image, a section of a mouse after induced pyramidal neuronal death is given [51]. (**B**, **C**) Representative images of microglia and astrocytes in corpus callosum (**B**) and hippocampal CA1 region (**C**), depicting prominent reactive gliosis in corpus callosum of *Cnp*^−/−^ mice, and virtually normal astrocytes and ramified microglia in the hippocampus; images acquired as 10 µm Z-stacks, displayed as maximum-intensity projections. (**D**, **E**) Representative images of amyloid precursor protein (APP)+ axonal swellings as neurodegeneration readout in corpus callosum (**D**) and hippocampal CA1 (**E**), illustrating axonal degeneration in *Cnp*^−/−^ mice. (**F**–**M**) Quantification of neuroinflammation (microgliosis, astrogliosis), axonal degeneration, and T cell infiltration in corpus callosum (**F**–**I**) and hippocampus (**J**–**M**) of *Cnp*^−/−^ mice with/without NR1-immunization versus NR1-immunized WT. Note the absence of any measurable influence of NMDAR1-AB. **N** Quantification of parvalbumin (PV)+ interneurons did not show differences between groups in hippocampus or hippocampal subregions; data from 5–6 mice/group; Welch’s ANOVA or Kruskal–Wallis test; two-tailed unpaired Welch’s corrected *t*-test or Mann–Whitney *U* tests; mean ± SEM presented.

When employing MWM, we found clear behavioral changes in classical hippocampal tasks, magnified by the presence of NMDAR1-AB. Thus, we first checked whether Fluorojade staining would reveal neurodegeneration in the hippocampus. Using this method, however, we saw overall intact hippocampal structures and cells (Fig. 3A). Overview images of gliosis (both micro- and astrogliosis) uncovered clear respective signals in corpus callosum of *Cnp*^−/−^ mice with or without NMDAR1-AB, but only weak signals in the hippocampus, consistent with the predominant white matter inflammation (Fig. 3B, C). Axonal degeneration, estimated by APP+ axonal swellings, was prominent both in callosal white matter tracts and hippocampus (Fig. 3D, E). The impressions gained by the overview images were subsequently confirmed by quantifications which additionally revealed T cell infiltration in *Cnp*^−/−^ mice, regardless of NMDAR1-AB presence (Fig. 3F–M). B cell numbers were very low and not different between groups (not shown). Interestingly, in contrast to the diphtheria toxin-induced gray matter encephalitis [24], parvalbumin+ interneurons in total hippocampus or its subregions, cornu ammonis and dentate gyrus, were not quantitatively changed in any of the conditions compared to WT (Fig. 3N), likely explained by the morphologically intact pyramidal layer (Fig. 3A). Together, these histological data emphasize once more the predominant white matter inflammation in the *Cnp*^−/−^ model with just moderate spreading of the inflammatory process to the hippocampus or other gray matter areas [32, 44]. The degenerative process, however, evaluated by APP+ axon counts, was clearly present in both compartments. Moreover, histological quantifications attest that NMDAR1-AB do not enhance the preexisting (before immunization) and progressing inflammatory condition. This obviously implies that the observed stair patterns of behavioral changes, with NMDAR1-AB playing an amplifier role, are due to an additional NMDAR1 dysfunction or downregulation by NMDAR1-AB rather than an exaggerated underlying encephalitis. Thus, NMDAR1-AB not only fail to induce an encephalitis on their own [9, 24, 59–63], but also to augment an underlying gray [24] or white matter inflammation as shown here. However, they can considerably shape the resulting behavioral phenotype. Of course, brain regions, other than those examined here, may well be involved in the behavioral NMDAR1-AB effects, too. Also, our findings with NMDAR1-AB, suggesting lack of any proinflammatory potential of these autoantibodies, may not necessarily be translatable to other conditions, e.g. systemic lupus erythematosus, where more toxic “autoantibody cousins,” targeting the NR2A and B subunits, may be at work [64–66].

### Genetic elimination of oligodendroglial NMDAR uncovers a potential contribution of these receptors to behavioral consequences of NMDAR1-AB

NMDA receptors are not only expressed by excitatory neurons but also by other cell types, like oligodendrocytes [67–69]. These in turn play a key role in regulating glucose uptake in response to axonal glutamate release upon neuronal activity, thus mediating metabolic support of axons on demand. Targeted inactivation of oligodendroglial NMDA receptors impairs axonal energy metabolism [47, 70]. So far, consequences of NMDAR downregulation by NMDAR1-AB in oligodendrocytes have only been addressed in vitro, showing robust downregulation of the glucose transporter GLUT1 and diminished Ca^2+^ responses upon NMDAR agonist application after NMDAR1-AB exposure [71]. Since in vivo aftereffects have remained unexplored, we wondered whether some of the impaired behavioral features, amplified by NMDAR1-AB in our model, might perhaps overlap with respective readouts measurable in mice with genetic elimination of oligodendroglial NMDAR. In other words, would such findings provide first hints of an involvement of oligodendroglial NMDAR in the overall consequences of NMDAR downregulation by NMDAR1-AB?

**Fig. 4:**
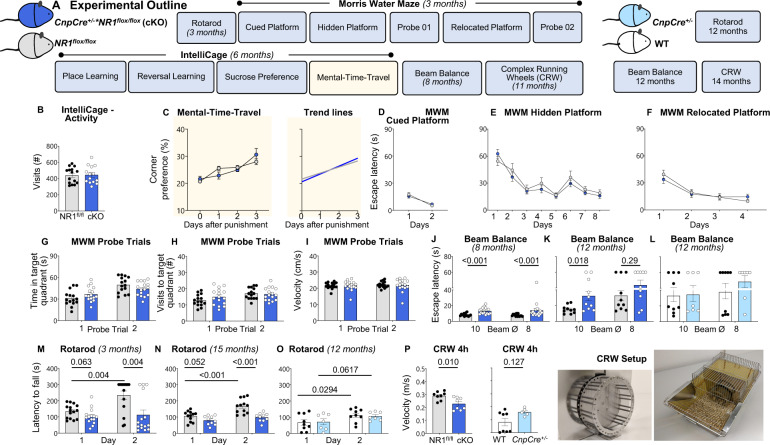
Behavioral characterization of mice lacking NR1 in oligodendrocytes suggests participation of oligodendroglial NMDAR in NMDAR1-AB effects. **A** Experimental outline of the study on female C57Bl/6 *CnpCre*^+/−^*:NR1*^*flox/flox*^ (cKO) mice and their *NR1*^*flox/flox*^ littermate controls (*n* = 16 each). Note that distinct behavioral paradigms were additionally tested in WT (*n* = 9) and *CnpCre*^+/−^ (*n* = 7) mice, to separate a potential impact of heterozygous *Cnp* deficiency from loss of oligodendroglial NR1. **B** Overall activity in IntelliCage paradigms and **C** MTT performance of cKO (*n* = 13–14) does not differ from *NR1*^*flox/flox*^ controls (*n* = 15), as demonstrated by trend lines (right), calculated by linear regression (GraphPad Prism 9 Software, San Diego, CA, USA). All slopes were non-zero (*p* < 0.001), documenting successful execution and MTT learning; similar MTT capabilities in cKO and control mice, p(slope)=0.345 (compare Fig. 1C, D). **D**–**I** Unaffected spatial memory and reversal learning in Morris water maze (MWM) during cued, hidden, and relocated platform training as well as during first and second probe trials. No differences were observed regarding the time spent in (**G**) or the number of visits to (**H**) the target quadrant. Overall locomotion was unaffected in cKO mice (**I**); *n* = 15 controls; *n* = 16 cKO; repeated measure mixed-model ANOVA revealed successful learning of the task, irrespective of genotype, with a significant main effect of time at all stages (*p* < 0.001). No significant time × genotype interaction was observed during the cued (*p* = 0.235), hidden (*p* = 0.654), or relocated (*p* = 0.211) platform training. **J** Motor coordination during beam balance is impaired in female cKO mice at the age of 8 months (*n* = 15) versus controls (*n* = 15); as well as **K** in a separate cohort of older females (12 months; *n* = 10 per genotype). **L** *CnpCre*^+/−^ mice are not affected (*n* = 9 and 7 for WT and *CnpCre*^+/−^, respectively). **M** Motor performance and learning in the rotarod is impaired in female cKO mice at the age of 3 months (*n* = 16 and 15 for control and cKO, respectively) as well as **N** in a separate cohort of aged females (*n* = 12 and 10, respectively). **O** Motor learning is unaffected in *CnpCre*^+/−^ mice; one-sided Welch’s corrected *t*-test. **P** Motor-cognitive abilities during 4 h of CRW (complex running wheel) performance is impaired in cKO mice but normal in *CnpCre*^+/−^; *n* = 8 per genotype; photographs of the CRW setup show omitted bars of CRW, demanding mice to adapt to irregular wheel pattern, i.e., requesting motor-cognitive performance; two-tailed unpaired Welch’s corrected *t*-test or Mann–Whitney *U* tests; mean ± SEM.

Thus, female conditional oligodendroglial NMDAR KO mice (*CnpCre*^+/−^*NR1^*flox/flox*^*)* went through an analogous test battery and were compared to *NR1*^*flox/flox*^ controls (Fig. 4A). These mice did not show any abnormalities in IntelliCage, including MTT, and MWM (Fig. 4B–I; Table 1). Interestingly, beam balance at 8 and 12 months of age, i.e. walking on 10 mm versus 8 mm diameter beams, revealed inferior escape latency in cKO females as compared to controls. Rotarod performance and, in particular, motor learning, defined as improvement from day 1 to day 2, was clearly compromised, as was the average velocity on CRW. Collectively, these data disclose a predominant motor/coordination inferiority of oligodendroglial NMDAR cKO mice. Reassuringly, these phenotypes were not observed in an extra control group of *CnpCre*^+/−^ and WT mice, employed to exclude effects of *Cnp* heterozygosity alone (Fig. 4J–P). Based on these observations, we speculate that the amplified MWM pathology in immunized *Cnp*^−/−^ mice (compare Fig. 1E–J) is explained mainly through downregulation by NMDAR1-AB of neuronal NMDAR, whereas their beam balance phenotype (compare Fig. 1K, L) may be contributed by the downregulation of oligodendroglial NMDAR. Even though this postulation is just indirect at this point, it may stimulate further research on a multicellular input to NMDAR1-AB mediated phenotypes.

## Conclusions and outlook

The present mild encephalitis model tested the hypothesis that high-level circulating NMDAR1-AB can shape the behavioral consequences of an underlying white matter inflammation in *Cnp*^−/−^ mice with confirmed BBB dysfunction. Whereas in the previously tested gray matter model [24], the magnitude of phenotype shaping was slight, it is relatively strong in the genetically induced, mild white matter encephalitis employed here, resulting in an intriguing stair pattern of behavioral pathology. However, also in white matter inflammation, NMDAR1-AB failed to further expand any histological readouts of inflammation. Together, these data indicate that the nature, location and degree of an underlying encephalitic process ultimately determine the clinical picture upon NMDAR1-AB exposure. This may also well be the case in the mild encephalitis forms typically diagnosed only postmortem in various neuropsychiatric conditions. At the same time, they explain the highly variable presenting symptoms as well as the different courses of “NMDAR encephalitis.”

## Data Availability

All data are available upon request.
